# Lipopolysaccharide-Induced Model of Neuroinflammation: Mechanisms of Action, Research Application and Future Directions for Its Use

**DOI:** 10.3390/molecules27175481

**Published:** 2022-08-26

**Authors:** Anna Skrzypczak-Wiercioch, Kinga Sałat

**Affiliations:** 1 Department of Animal Anatomy and Preclinical Sciences, University Centre of Veterinary Medicine JU-UA, University of Agriculture in Krakow, 24/28 Mickiewicza St., 30-059 Krakow, Poland; 2 Department of Pharmacodynamics, Chair of Pharmacodynamics, Jagiellonian University Medical College, 9 Medyczna St., 30-688 Krakow, Poland

**Keywords:** lipopolysaccharide, neuroinflammation, neurodegenerative diseases, Alzheimer’s disease, Toll-like receptor 4

## Abstract

Despite advances in antimicrobial and anti-inflammatory therapies, inflammation and its consequences still remain a significant problem in medicine. Acute inflammatory responses are responsible for directly life-threating conditions such as septic shock; on the other hand, chronic inflammation can cause degeneration of body tissues leading to severe impairment of their function. Neuroinflammation is defined as an inflammatory response in the central nervous system involving microglia, astrocytes, and cytokines including chemokines. It is considered an important cause of neurodegerative diseases, such as Alzheimer’s disease, Parkinson’s disease and amyotrophic lateral sclerosis. Lipopolysaccharide (LPS) is a strong immunogenic particle present in the outer membrane of Gram-negative bacteria. It is a major triggering factor for the inflammatory cascade in response to a Gram-negative bacteria infection. The use of LPS as a strong pro-inflammatory agent is a well-known model of inflammation applied in both in vivo and in vitro studies. This review offers a summary of the pathogenesis associated with LPS exposure, especially in the field of neuroinflammation. Moreover, we analyzed different in vivo LPS models utilized in the area of neuroscience. This paper presents recent knowledge and is focused on new insights in the LPS experimental model.

## 1. Introduction

Neuroinflammation is defined as an inflammatory response in the central nervous system (CNS), mediated by production of cytokines together with chemokines, and inflammatory enzymes. CNS, as a structure separated by the blood–brain barrier (BBB), is equipped with resident immunocompetent cells, namely microglia and astrocytes. Microglia are a type of glial cells related to macrophages that constitute the main pool of immune cells within the brain and spinal cord. Microglia plays a crucial role in maintaining homeostasis in the nervous tissue of the CNS by sensing and eliminating unnecessary metabolic products, foreign materials and cellular debris. For this reason it is sometimes known as “housekeeping cells” [1]. However, it has been proven that role of microglia exceeds beyond housekeeping, as it participates in the brain development, neuromodulation, synaptic plasticity, and it contributes to learning and memory processing [2,3]. Another group of CNS cells that possess immunological properties are astrocytes. Similarl to microglia, astrocytes have many different functions. They are essential for both the developing and adult brain, and their most prominent role is to maintain BBB [4]. However, as mentioned above, both microglia and astrocytes are immunocompetent cells, and they play a pivotal role in the neuroinflammation [5]. They express membrane receptors and molecules, such as the receptor for advanced glycation end-products (RAGE), clusters of differentiation-36 (CD-36), scavenger receptors (SRs), Fc receptors, complement receptors (CRs), and Toll-like receptors (TLRs), to detect aggression factors and homeostasis imbalance [6]. Focusing on the TLRs family, the microglial cells express TLR4 and TLR2 and their activation has been associated with neuroinflammation and extensive neuronal cell death [7,8]. Infection, stroke, traumatic brain injury, neurodegeneration and cancer reactivate these groups of cells, promoting pro-inflammatory changes in their morphology along with the production of inflammatory mediators [2]. Upon reactivation, microglial cells transform into pro-inflammatory phenotype M1 and start to produce pro-inflammatory cytokines, including tumor necrosis factor-α (TNF-α), interleukin (IL)-1β, IL-6, IL-12, IL-1α, complement component 1q (C1q), prostaglandin E2 (PGE_2_) and free radicals, namely nitric oxide (NO) and reactive oxygen species (ROS), to provide protection against harmful factors [7,9]. Interestingly, in addition to pro-inflammatory factors, stimulated microglia also produce IL-10, which is a potent anti-inflammatory cytokine. During early stages of neuroinflammation, only small quantities of IL-10 can be detected, but its influence on modulation over the inflammatory response at this point is crucial. Microglial production of IL-10 increases over time, and plays a role in a resolution of inflammation [10]. Prolonged reactivation of microglia results in an increase of oxidative and nitrosative stress and mitochondrial dysfunction, which leads to neuronal death and neurodegeneration, especially in the hippocampal area [1,8,11,12]. In addition, Liddelow and colleagues showed that chronic microglial-derived pro-inflammatory stimuli, including TNF-α, IL-1α and C1q, led to transformation of resting astrocytes into neurotoxic reactive astrocytes, responsible for killing neurons and differentiated oligodendrocytes [13]. Due to their strong immunogenic potential, microglia and astrocytes play a key role in the neuroinflammation. Their physiological activity is crucial for maintaining homeostasis and providing precise response to aggression factors; however, their uncontrolled and excessive reactivation is neurotoxic. 

Neuroinflammation has been associated with several neurological diseases, such as Alzheimer’s disease (AD), Parkinson’s disease (PD), amyotrophic lateral sclerosis, multiple sclerosis, Huntington’s disease, and brain cancer [1,2,4,9,14]. These neurodegenerative diseases and their complications are a grave concern for public health given their incurability, reduction in patient’s quality of life and self-independence, and the increasing number of cases every year [15]. For example, in 2015 dementia affected 47 million of people worldwide, and AD is the estimated cause of 60-70% of these cases, according to World Health Organization (WHO) [16]. Moreover, AD is the sixth most common cause of death amongst the elderly population [17]. Despite this, exact understanding of the pathogenesis and risk factors of these diseases still remains incomplete [8]. The vast majority of currently available therapeutic options can only reduce the symptoms associated with the illness and cannot prevent or inhibit the development of the disease. To date, five of six drugs registered in human use for AD provide only symptomatic treatment [15,17]. In the 2021 a novel monoclonal antibody, namely aducanumab, was approved by the Food and Drug Administration (FDA) as the first therapeutic agent affecting AD’s pathomechanism by reducing amyloid-β (Aβ) plaques formation in the brain [15]. However, aducanumab cannot completely cure AD and it is not suitable for many individuals, including patients with advanced AD [15]. In addition, there seems to be insufficient data documenting the efficacy and safety profile of aducanumab in AD patients, which raises serious concerns about its use [18]. PD is also a widespread neurological disease. The WHO expert opinion estimates the number of patients with PD in 2019 at over 8.5 million, which makes it the second most common progressive neurodegenerative disease worldwide after AD [19]. Disability and death related to PD are also a serious and growing problem in the aging society. Current treatment strategies of PD include drugs enhancing the dopaminergic neurotransmission in the brain, namely levodopa, a precursor of dopamine, used in combination with inhibitors of enzymes peripherally metabolizing levodopa, and dopaminergic receptor agonists, e.g., pramipexole. Another group of drugs reducing PD symptoms are muscarinic receptor antagonists, cholinesterase inhibitors, N-methyl-D-aspartate (NMDA) receptor antagonists, and adenosine A_2A_ receptor antagonist [20]. Similarl to AD, pharmacological treatment remains symptomatic, without ability to cease disease progression. All of this creates an urgent need to develop effective and safe disease-modifying therapies.

Accumulation of extracellular plaques of Aβ peptides and intracellular neurofibrillary tangles (NFT) consisting of tau protein in the CNS are hallmark features of AD [21]. Aggregates of these aberrant proteins trigger the inflammatory response, causing microglial cell reactivation and astrogliosis [17,22]. Microglia recognizes Aβ mainly by TLR2 and TLR4, and Aβ-stimulation can induce dual activity in these cells. At early stages, the communication between neurons, microglia and astrocytes occurs, mediated via the neuronal Aβ-astrocytic C3/C3a–microglial C3aR axis [2]. This intercellular communication leads to release of neuroprotective cytokines, neurotrophic factors, neurotransmitters, and intensification of Aβ phagocytosis in order to remove harmful amyloid aggregates [21]. However, in the AD these innate protective mechanisms are not sufficient to remove Aβ plaques and restore homeostasis. Consequently, the process becomes chronic, and constant stimulation of microglia by Aβ provokes their cytotoxic properties [2,21] and may cause TLR4 overexpression [22]. On the other hand, it has been suggested that chronic neuroinflammation may be present even before the appearance of Aβ pathologies in the late-onset AD [23], which raises questions about other pro-inflammatory factors involved in development of AD.

Increasing evidence proves that neuroinflammation is also a major cause involved in the pathogenesis of PD and its clinical presentation [9,24]. In PD, progressive degeneration of dopaminergic neurons in the substantia nigra pars compacta (SNpc) occurs along with the accumulation of α-synuclein aggregates (Lewy bodies) within survived neurons. Abnormally deposited α-synuclein stimulates the neuroinflammation by activating microglia [9]. Positron emission tomography and post-mortem analysis in PD patients indicated increased reactivation of microglia, followed by elevated pro-inflammatory cytokine levels in serum, cerebrospinal fluid and SNpc compared to neurologically healthy patients, which was demonstrated in studies of Surendranathan and colleagues [25], Lavisse and colleagues [26], and was summarized in a review by Deng and colleagues [9]. It is believed that microglial overreactivity is triggered directly by α-synuclein, which results in the clinical onset of the disease and its progression [27]. TLR4 may be also involved in a microglial recognition of α-synuclein aggregates and triggering the inflammatory cascade [9,24]. Moreover, a study by Kouli and colleagues showed elevated expression of TLR4 and increased IL-1β levels in regions critically associated with PD in brains of deceased patients with PD dementia [24]. Astrocytes also express TLR4; however, involvement of this pathway into α-synuclein response is inconclusive. On the one hand, endocytosis of α-synuclein can occur by a TLR4-independent pathway, but on the other hand high concentrations of extracellular α-synuclein can trigger a TLR4-dependent inflammatory response. The threshold between scavenging endocytosis and inflammation in reaction to the α-synuclein has not been established yet, so further research in this matter is needed. It is known, however, that the accumulation of a large amount of α-synuclein and inflammation in the astrocytes causes impairment of their function, including dysregulation in the maintenance of BBB [4].

Taken together, neuroinflammation is an inseparable element of discussed neurodegenerative diseases. To date, the main common point between neuroinflammation occurring in the course of these diseases and LPS-induced inflammation is a pronounced activation of TLR4-signaling and a production of potent pro-inflammatory cytokines. To model the complexity of processes involved in the neuroinflammation and their outcomes, e.g., cognitive dysfunction, in vivo studies are intensively utilized. For this purpose, LPS is used as one of the most potent pro-inflammatory stimuli [14,28]. 

## 2. Lipopolysaccharide—Insights in the Structure

**Figure 1:**
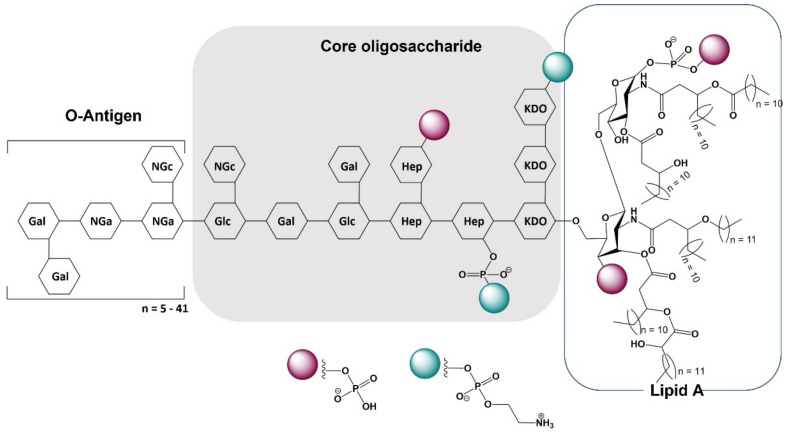
General structure of lipopolysaccharide from *E. coli* O111:B4. Abbreviations: Gal-galactose; Glc-glucose; Hep-L-glycerol-D-manno-heptose; KDO-2-keto-3-deoxyoctonic acid; Nga-N-acetyl-galactosamine; NGc-N-acetyl-glucosamine.

Lipopolysaccharide (LPS), commonly known as endotoxin, is a major component of the outer membrane of Gram-negative bacteria that induces a pronounced inflammatory response. LPS is a heat-stable glycolipid that serves as a vital reinforcement of the outer membrane’s asymmetric phospholipid bilayer, providing structural integrity and permeability barrier to a bacterial cell [29]. The LPS molecule consists of lipid A, core oligosaccharide and O-polysaccharide also referred to as O-antigen (Figure 1). Hydrophobic lipid A is the most immunogenic part of the LPS, embedded in the bacterial outer membrane and merging it with the rest of the LPS molecule [30]. Lipid A is the most stable component of LPS, as O-oligosaccharide and core moieties may vary significantly even among the same bacteria species. However, even in the conservative lipid A, slight chemical differences can occur like a variability in acylation and phosphorylation levels, which can result in a diverse immunogenicity of LPS. The influence of the lipid A structure on LPS immunogenicity is well-described and currently it is believed that a six-acyl chain (hexa-acylated) lipid A is the most immunogenic form of LPS [29,30], [31]. This relationship provides a fundamental base for immune distinguish between pathogenic and non-pathogenic bacteria [32]. The hexa-acylated variant of lipid A is found in potent pathogens like *Escherichia coli* and *Salmonella enterica* serovar *Typhimurium* [33]. Nevertheless, some bacteria have developed strategies to elude the immune recognition of hexa-acylated lipid A and increase survival in the host’s environment. These mechanisms include modifications of the lipid A by adding 4-amino-arabinose (Ara4N) residues or ethanolamine to phosphate groups, removing phosphate groups, or finally, by shortening or lengthening of acyl chains [30,34]. For example, virulent Gram-negative bacteria *Yersinia pestis* and *Yersinia pseudotuberculosis* produce highly immunogenic hexa-acylated lipid A in lower ambient temperatures, but at the temperature of a mammalian body, namely 37 °C, the less active tetra-acylated form is expressed [30]. 

Living organisms are constantly exposed to pathogenic and non-pathogenic bacteria. Gram-negative bacteria are the cause of many serious infectious diseases in both humans and animals, such as salmonellosis, colibacteriosis, brucellosis and other. The innate, or nonspecific, immunity is a first line of vertebrates’ defense against bacterial infiltration, and it acts as physical, chemical and cellular barrier to infection. Innate immune cells include neutrophils, macrophages, dendritic cells, and natural killer cells [35]. To detect harmful factors, immune cells are equipped with pattern recognition receptors (PRRs) that comprise TLRs, NOD-like receptors (NLRs) and retinoic acid inducible gene-I-like receptors (RLRs). PRRs become activated upon recognition of pathogen-associated molecular patterns (PAMPs) which include intact pathogens or their components, such as LPS, and damage-associated molecular patterns (DAMPs) containing host’s cells molecules related to cellular damage or death. Binding a ligand to PRR initiates a downstream signaling pathway that triggers inflammatory cascade [36]. The LPS molecule is a potent PAMP, stimulating a pronounced inflammatory response; the mode of its detection by the immune system is discussed below.

## 3. Cellular Recognition of LPS

### 3.1. Toll-Like Receptor 4

Toll-like receptors (TLRs) are a class of PRR which recognize aggression factors and provide rapid inflammatory responses to them. TLRs are found in innate immune cells and some non-immune cells. In the CNS these receptors are primarily expressed on microglia and astrocytes, but they can be found also on oligodendrocytes, neurons and Schwann cells [37]. TLRs recognize a wide array of PAMPs and DAMPs, including, inter alia, bacterial, viral, fungal and protozoan components, heat shock proteins, hyaluronian fragments, ATP or the host’s DNA. In humans, 10 subtypes of TLRs have been characterized (TLR1-10), with 12 in mice (TLR1-9, TLR11-13). These subtypes differ in ligand specifity, cellular localization, mode of multimerization upon activation and downstream signaling pathway. They can be localized on the surface of the cell (TLR1, 2, 4, 6, 10) or in intracellular compartments (TLR3, 7, 8, 9, 11, 12, 13) [36]. In brief, TLR-signaling induces recruitment of specific adaptor molecules, leading to activation of the transcription factors, which stimulate production of inflammatory mediators, including cytokines and chemokines, such as TNF-α, IL-1α, IL-1β, IL-6, IL-8, IL-12, IL-23, macrophage inflammatory protein (MIP)-1α, and MIP-1β [37,38,39].

TLRs sense PAMPs and DAMPs through their outer leucine-rich repeat domain (LRR). Upon ligand binding, this ectodomain undergoes homo- or heterodimerization, activating intracellular TLR’s Toll/IL-1 receptor domain (TIR) which induces a downstream signaling pathway [39]. It starts with recruitment of intracellular adaptor proteins, namely myeloid differentiation primary response protein (MyD88), MyD88-adopter-like protein (MAL, also known as TIR domain-containing adaptor protein- TIRAP), Toll/interleukin-1-receptor domain-containing adaptor-inducing interferon β (TRIF) and TRIF-related adaptor molecule (TRAM) [38]. TLRs are localized on the cell surface signal through MyD88, which binds to TLR’s domain via MAL/TIRAP protein. Intracellular TLRs, except for TLR3 and endosomal TLR4, interacts directly with MyD88 without participation of MAL/TIRAP. This process is called MyD88-dependent signaling pathway. The complex of MyD88 with TLR induces attachment of interleukin-1 receptor-associated kinases (IRAKs), including IRAK4 and IRAK 1/2. They form together an oligomeric structure called a Myddosome [40], and in this particular conformation IRAKs become phosphorylated. Subsequently, activated IRAKs recruit TNF-receptor-associated factor 6 (TRAF6) to the Myddosome, which stimulates the transforming growth factor β-activated kinase 1 (TAK1) and TAK1-binding protein 1 and 2/3 (TAB1, TAB2/3). This process activates Iκβ kinase complex (IKK) consisting of IKKα, IKKβ, IKKγ and their regulatory subunit NF-κB essential modulator (NEMO) along with activation of mitogen-activated protein kinases (MAPKs; including ERK1/2, JNK and p38). IKKs and MAPKs activate transcription factors: nuclear factor-κB (NF-κB) and activator protein-1 (AP-1), respectively. NF-κB and AP-1 translocate to the nucleus, where they trigger the production of pro-inflammatory mediators, such as IL-6, TNF-α and enzymes, inter alia, inducible nitric oxide synthase (iNOS) and cyclooxygenase-2 (COX-2) [36,37,38]. 

On the other hand, remaining endosomal TLR3 and TLR4 induce TRIF-dependent signaling cascade. TLR3 can recruit TRIF directly, while internalized TLR4 requires adaptor protein TRAM to bind with TRIF. Activated TRIF recruits TNF receptor-associated factor 3 (TRAF3), which further activates serine/threonine-protein TANK-binding kinase 1 (TBK1) and IKK inducible kinase (IKKi). They stimulate phosphorylation of interferon regulatory factors 3 and 7 (IRF3 and IRF7) and then, IRF3/7 dimerize and translocate to the nucleus, where they induce production of type I interferons. Moreover, TRAM-TRIF complex created with endosomal TLR4 can also interact with TRAF6 via receptor-interacting serine/threonine-protein kinase 1 (RIP1 also referred as RIPK1), triggering a pathway leading to activation of NF-κB and AP-1 [38,40].

A complete list of known TLR ligands can be found elsewhere [37,40]. For the purposes of this article, we will focus exclusively on LPS-recognizing receptor, namely the TLR4. TLR4s are expressed in many immune and non-immune mammalian cells, including macrophages, monocytes, peripheral blood lymphocytes, granulocytes, dendritic cells, microglia, astrocytes, brain endothelial cells, dermal micro-vessel endothelium, umbilical vein endothelium and adipocytes [32]. However, in the CNS, the largest pool of TLR4 is associated mainly with microglial cells [22]. In order for TLR4 to bind the ligand, LPS must be presented in a certain manner. Initially, circulating LPS molecules or intact Gram-negative bacteria get caught by a special acute-phase protein called lipopolysaccharide-binding protein (LBP) that senses the lipid A motif. Subsequently, LPS is transferred to the cluster of differentiation-14 (CD-14) that presents the molecule to TLR4. However, LPS is not bound directly by the LRR region of TLR4, but through a hydrophobic cleft in the accessory protein MD-2 that is associated with TLR4’s LRR [35]. Upon LPS binding, homodimerization of TLR4 takes place, activating a downstream signaling pathway. TLR4 is unique because it can signal by both MyD88- and TRIF-dependent pathways [38]. When TLR4 is expressed on the cell surface, MyD88-dependent signaling occurs. However, TLR4 also has the ability to be endocytosed upon ligand binding [39]. After internalization into endosome, TLR4 signals via a TRIF-dependent signaling cascade which moreover can reinforce NF-κB and AP-1 response. This dual activation pathway mutually complements and intensifies its action, providing potent pro-inflammatory reaction to bacterial infection. The final result of TLR4 activation is a production of cytokines and chemokines, including IL-1β, IL-6, IL-8, TNF-α, PGE_2_, ROS, NO and type I interferons [1], [22,41].

However, studies over the last decade have revealed that there are also other than TLR4 pathways participating in LPS detection. To the date, two TLR-independent LPS recognition systems are known, including transient receptor potential (TRP) channels and inflammatory caspases [33,42].

### 3.2. TLR4-Independent Pathways of LPS Detection

The observation of some rapid, painful effects of LPS, such as tail-flick hyperalgesia following intraperitoneal administration of LPS, could not been explained by a TLR4-dependent inflammatory cascade, and this led to further research in this field. This phenomenon has been explained by the discovery of transient receptor potential (TRP) channels sensitive to LPS [33,43]. TRP channels are a superfamily of ion channels. These proteins are present in a variety of cells and are an important element of maintaining cellular homeostasis. The TRP channels family comprises two groups and seven subfamilies, divided in accordance with the channel structure. They differ in the ligand specificity and the selectivity of the ion influx. LPS-responsive TRP channels include transient receptor potential ankyrin 1 (TRPA1), transient receptor potential vanilloid 4 (TRPV4), transient receptor potential cation channel subfamily V member 1 (TRPV1), transient receptor potential melastatin 3 and 8 (TRPM3 and TRPM8, respectively) [44]. TRPA1 channels are predominantly found on the surface of sensory neurons; their activation is responsible for sensations of pain and cold, and then a development of neurogenic inflammation via Ca^2+^ influx, release of calcitonin gene-related peptide (CGRP) and pro-inflammatory sensory neuropeptides, e.g., substance P (SP) [45]. It explains fast nociceptive reactions to LPS [33]. Interestingly, CGRP demonstrated an inhibitory effect on neutrophils and macrophage functions [46], and this relationship seems to deserve a closer look in the context of LPS-induced neuroinflammation. However, studies using knockout trpa1−/− mice showed reduction in inflammation, preliminarily negating this hypothesis [47]. On the other hand, the study by Hajna and colleagues [48], focused on the airway inflammation, presented a complex role for TRPA1 channels in the inflammatory response. Its results showed protective function of TRPA1 channels in acute LPS-induced airway inflammation, but not in the chronic inflammation induced by a cigarette smoke. In this study, trpa1−/− mice were also an animal model. Taken together, the role of TRPA1 in LPS-induced inflammation is not fully explained yet. TRPV1 is another cation channel involved in the nociception and it is often co-expressed with TRPA1 on sensory neurons [48]. Its activation leads to the release of CGRP, SP and somatostatin, similarly to TRPA1. Despite some controversy, the expression of TRPV1 has been also shown on neurons and microglia in the CNS, where it may modulate the neuroinflammatory processes; however, an exact underlying mechanism of this relationship is not understood yet [49]. TRPV1 channels have been hypothesized as a neuroprotective target in neurodegenerative diseases, such as AD and PD. Pharmacological activation of TRPV1 by capsaicin showed protective effects a in transgenic mouse model of AD [50,51] and in the PD mouse model [52]. Here appears another unanswered question about the relationship between LPS and TRPV1. LPS is a potent neurodegenerative agent itself, but also a ligand for TRPV1, which on the contrary, can alleviate neuroinflammation. Defining a role for LPS-stimulated TRPV1 activation in the LPS-induced neuroinflammation seems to be a compelling scientific goal. TRPV4 participates in an early detection of LPS in epithelial cells, which has been particularly well-studied in murine airway epithelial cells and in macrophages [33]. Similarly, TRPA1 and TRPV1, and TRPV4 channels have also been detected in the CNS. This is particularly the case with the high expression of TRPV4 which occurs in astrocytes [53]. However, the influence of TRPV4 on inflammation tends to be clearer. To the date, it is established that the activation of TRPV4 exacerbates inflammatory responses and triggers neuroinflammation, while TRPV4 inhibitors exert a neuroprotective effect [53,54]. TRPV4 is a nonselective Ca^2+^ channel, and its activation results in an increased intracellular Ca^2+^ concentration and increased iNOS activity, which leads to the production of pro-inflammatory mediators, such as eicosanoids [55]. However, precise mechanisms for cellular signaling triggered by TRPV4 are not fully explained and require further research. Remaining TRP channels sensitive to LPS, namely TRPM3 and TRPM8, are not so thoroughly studied yet and, to date, their role in the LPS response remains unclear. 

An intracellular recognition of LPS is provided by specialized inflammatory caspases, namely caspase 4 and 5 in humans, and caspase 11 in mice, and this signaling pathway is also being called a ‘non-canonical’ inflammasome activation [33]. Caspases are a family of cysteine proteases playing an essential role in programmed cell death and innate immunity, and human caspase 4 and 5 and their murine counterpart, caspase 11, are able to directly sense LPS [56]. The above-mentioned caspases recognize lipid A motif via caspase recruitment domain (CARD). Upon LPS recognition in the cytoplasmatic milieu it comes to caspase oligomerization and cleavage of gasdermin-D (GSDMD), which permeabilizes cell membrane and induces pyroptosis [57]. Moreover, it has been presented that triggered caspases of a ‘non-canonical’ pathway can promote the activation of the ‘canonical’ NLR family pyrin domain-containing-3 (NLRP3) inflammasome and thus induce an inflammatory response, resulting in production of IL-1β and IL-18 [58]. Wang and colleagues also demonstrated increased levels of activated caspase-11 and NLRP3 in LPS-stimulated murine primary astrocytes [59].

**Figure 2:**
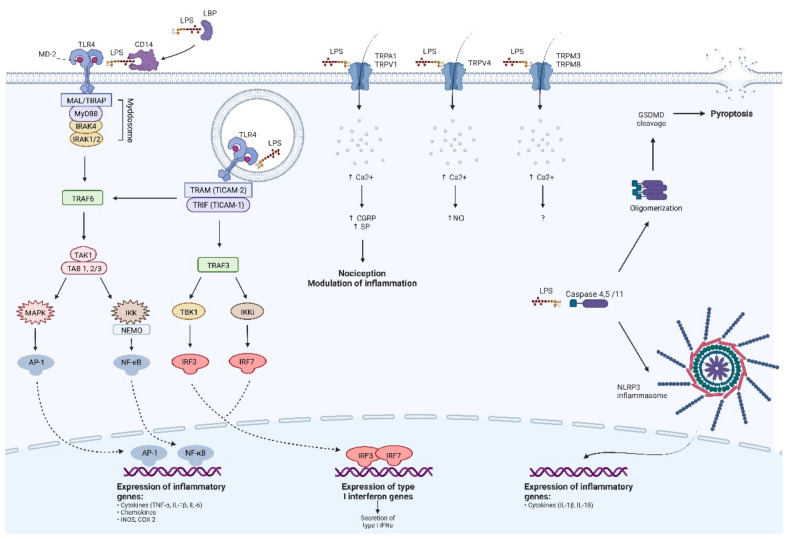
Mechanisms of molecular response to LPS. A detailed description can be found in the section ‘Cellular recognition of LPS’. Abbreviations: AP-1—activator protein-1; CD14—cluster of differentiation-14; CGRP—calcitonin gene-related peptide; COX-2—cyclooxygenase-2; GSDMD— gasdermin-D; IFN—interferon; IKK—Iκβ kinase complex; IKKi—IKK inducible kinase; IL—interleukin; iNOS—inducible nitric oxide synthase; IRAK—interleukin-1 receptor associated kinase; IRF—interferon regulatory factor; LBP—lipopolysaccharide-binding protein; LPS—lipopolysaccharide; MAL—MyD88—adopter-like protein; MAPK—mitogen-activated protein kinase; MD-2—accessory protein MD-2; MyD88—myeloid differentiation primary response protein; NLRP3—NLR family pyrin domain containing 3; NEMO—NF-κB essential modulator; NF-κB—nuclear factor-κB; NO—nitric oxide; SP—substance P; TAB—TAK1 binding protein; TAK1—transforming growth factor β-activated kinase 1; TBK1—TANK-binding kinase 1; TIR—Toll/IL-1 receptor domain; TIRAP—TIR domain containing adaptor protein; TNF-α—tumor necrosis factor-α; TLR4 —Toll-like receptor 4; TRAF—TNF-receptor associated factor; TRAM—TRIF-related adaptor molecule; TRIF—Toll/interleukin-1-receptor domain-containing adaptor inducing interferon β; TRPA1—transient receptor potential ankyrin 1; TRPM3—transient receptor potential melastatin 3; TRPM8—transient receptor potential melastatin 8; TRPV1—transient receptor potential cation channel subfamily V member 1; TRPV4 - transient receptor potential vanilloid 4. Adapted from ‘TLR Signaling Pathway’, by BioRender.com (2022). Retrieved from https://app.biorender.com/biorender-templates (accessed on 15 July 2022).

Summarized pathways of LPS cellular detection are presented below (Figure 2).

## 4. The Utility of LPS Inflammation Model in the Research Field

Powerful pro-inflammatory properties and well-studied TLR4-dependent mechanisms of immune system activation resulted in the extensive use of LPS as a model of sepsis and inflammation in mammals [60,61]. Nowadays, advanced medical and veterinary studies utilize more specific in vitro and in vivo models of LPS-induced oxidative stress and inflammation, for example LPS-induced acute lung injury [62], cardiac dysfunction [63], periodontitis [64], acute septic renal injury [65], uveitis [66], mastitis [67], endometritis [68], preeclampsia [69], and other. However, there is a particularly wide use of the LPS model in the area of neuroscience research. Neuroinflammation is an important pathomechanism associated with neurodegenerative diseases, such as AD and PD. 

A pivotal common point between neuroinflammation and LPS-induced inflammation is the TLR4 signaling pathway. The administration of LPS causes inflammatory response mediated mainly by TLR4, microglial reactivation and neuroinflammation, which results in the degeneration of neurons, synaptic loss and finally neuronal cell death [70]. This process is mediated by production of inflammatory molecules, such as TNF-α, IL-6, and IL-1β, increased activity of iNOS, COX-2, β-secretase, γ-secretase, Aβ accumulation and oxidative stress [70,71]. Amyloidogenesis caused by LPS is the most prominent phenomenon in the cortical and hippocampal areas [72]. Subsequently, in in vivo conditions, cognitive impairment occurs, followed by changes in normal behavior including decreased locomotion, anxiety, depression, somnolence, decreased appetite and weight loss, also known as sickness behaviors [1]. In patients suffering from neurodegenerative diseases, similar pathomorphological and clinical symptoms are found. Increased Aβ accumulation due to chronic LPS administration makes the LPS model a very adequate method for AD studies [71]. LPS can also induce characteristic features of PD by causing neuroinflammation and dopaminergic neuron loss [73,74]. The application of LPS experimental models in amyotrophic lateral sclerosis and Huntington’s disease was presented in an excellent review by Batista and colleagues [75]. However, the limitations of the LPS model must also be taken into consideration. First and foremost, the complexity of micro- and macromechanisms and crosstalks in neurogenerative diseases cannot be completely recreated by LPS-induced in vitro and animal models. For example, there is a difference in a ratio of TLR4 expression in microglia and astrocytes between humans and mice, which may influence the contribution of individual cell types in the neuroinflammation [22]. For this reason, the LPS models are a good research tools for studying anti-inflammatory and neuroprotective activities; however, a direct translation of in vitro or in vivo results to clinical application is not possible.

## 5. Technical Aspects of LPS Model Execution—Focus on AD and PD Modeling 

As mentioned above, the administration of LPS is commonly used to study neuroinflammation-associated diseases. However, many study designs differ from each other in LPS dose, frequency and route of administration. Therefore, here we compared a variety of LPS-induced neuroinflammation models and their outcomes in rodents. For this purpose, we reviewed articles published from 2015 to 2022 using PubMed, Google Scholar and Scopus, related to keywords ‘neuroinflammation’, ‘LPS’, ‘Alzheimer’s disease’, and ‘Parkinson’s disease’. To induce neuroinflammation in the animal model, LPS can be administered intraperitoneally, stereotaxically and intranasally [76]. A summary of experimental designs carried out in analyzed research papers is demonstrated in the Table 1.

Intraperitoneal injection of LPS is the most common route of systemic administration of LPS. Zhao and colleagues also noted the differences in carrying out the LPS model of neuroinflammation, hence they applied both intraperitoneal and intracerebroventricular administration of LPS in their research [1]. Mice receiving LPS intraperitoneally were divided further into two groups according to LPS dose 500 μg/kg or 750 μg/kg. The intraperitoneal injections of LPS were performed for seven consecutive days, 6 h before running the tests. The intracerebroventricular administration of 12 μg of LPS in 3 μL of saline was performed once using stereotaxic coordinates adapted from Haley and McCormick. Cognitive impairment induced by LPS was assessed in the Morris water maze test (MWM) and passive avoidance test (PAT). In MWM, all groups receiving LPS had a significantly longer latency time since day 2 of the testing trial. In the probe trial, the group treated with 750 μg/kg LPS intraperitoneally stayed in the platform quadrant for the shortest time, while the group with LPS administered intracerebroventricularly made the fewest crossings through the platform’s place. In the PAT, groups receiving LPS stayed in the illuminated compartment for a shorter period of time than groups receiving saline. Results obtained in both of these tests showed that each performed LPS administration regimen was effective at inducing cognitive impairment in mice. However, LPS also influenced motor coordination in the pole test (PT). LPS-induced mice had lower motor coordination scores compared to animals in control groups. Immunofluorescence staining of critical regions of hippocampus in sacrificed mice has further revealed microglia reactivation and loss of neurons in the LPS groups [1]. 

Recently, Alzahrani and colleagues directly addressed the problem of LPS dosage in the chronic intraperitoneal administration model of memory impairment [14]. Based on previous publications in this field, they investigated the influence of 7 days treatment with 250, 500 and 750 μg/kg LPS on recognition memory impairment in mice. They used the open field task (OFT) to assess locomotor activity and the novel object recognition task (NOR), the novel arm discrimination task (NAD) to examine short-term and spatial memory, respectively. In addition, ex vivo histological studies of the brain were performed. These studies demonstrated that only the highest dose (750 μg/kg) of LPS impaired both short-term and spatial memory. Based on the obtained results, it has been hypothesized that doses of 250 and 500 μg/kg LPS are likely to be associated mainly with hippocampal dysfunction, whereas the dose of 750 μg/kg can induce both the hippocampal and prefrontal cortex damage. However, in another study LPS administered intraperitoneally for 7 days at the dose of 250 μg/kg resulted in significant differences between control and LPS-treated groups in NOR [23,77]. This dissimilarity may have arisen from different data processing, and in the case of study by Fronza and colleagues [77] from the use of distinct type of LPS, which is discussed later in this section.

In another study focusing on neuroinflammation and conducted by Yang and colleagues [70], LPS was administered to mice intraperitoneally at the dose of 1 mg/kg, once daily for 5 days in order to induce chronic neuroinflammation. This method of administration of LPS at a higher dose but for a shorter time was also effective in producing chronic neuroinflammation in mice. Similar to previous studies, LPS was administered 6 h before the start of the tasks.

Cognitive impairment can be induced even after a single systemic injection of LPS [80]; however, this is believed to be associated with sickness behavior following acute inflammation rather than pathomorphological changes in the brain [23]. On the other hand, there are papers reporting persistent changes in the brain and cognition upon single LPS administration, even after 10 months after LPS injection [80]; this is consistent with the very first report of neurotoxicity after LPS exposure, when a laboratory worker developed PD-like signs 3 weeks after exposure to 10 μg of LPS from *Salmonella minnesota* through an open wound [9]. Currently, a single systemic injection of LPS at dose 5 mg/kg is also considered a reliable method of inducing acute and chronic neuroinflammation. A thorough elucidation of the effect of a single systemic injection of LPS on neuroinflammation was provided in an excellent article of Zhao and colleagues [81]. This work showed that a single intraperitoneal injection of LPS at the dose of 5 mg/kg was effective in inducing acute, and subsequently chronic neuroinflammation, in contrast to the lower dose of 1 mg/kg which was only able to induce the acute phase. The reason for this discrepancy may be the involvement of NLRP3-IL-1β-IL1R1 axis. A series of tests, including inter alia tyrosine hydroxylase immunohistochemistry and immunofluorescence showed gradual loss of dopaminergic neurons within substantia nigra (SN) upon depostion of 5 mg/kg LPS. This makes this model a relevant method for PD studies, especially in the field of a phase transition of neuroinflammation. In the aforementioned study by Yang and colleagues [70], a single intraperitoneal injection of 5 mg/kg LPS was utilized to induce acute model of neuroinflammation. 

An interesting aspect of intraperitoneal administration of LPS is how it reaches the CNS upon deposition in the peritoneal cavity. Initially, it has been suggested that LPS cannot cross BBB and neuroinflammation is stimulated by the cytokine and chemokine storm produced peripherally. However, the naturalistic study by Vargas-Caraveo and colleagues presented that under physiological conditions small amounts of LPS are detectable in blood and also in parts of brain separated by BBB [82]. The passage of LPS or its immunogenic part (lipid A) most likely occurs via transportation with LBP and in conjunction with lipoproteins [82]. Moreover, the high concentrations of LPS like the ones achieved in the LPS-neuroinflammation model can disrupt BBB, which occurs through various mechanisms [83,84]. The impaired BBB passes a greater amount of LPS along with inflammatory cells and mediators from the circulation to the brain, leading to substantial exacerbation of neuroinflammation.

The intraperitoneal route of LPS administration is one of the simplest methods to induce LPS-induced neuroinflammation. Performing an intraperitoneal injection is relatively easy and convinient for researchers carrying out the procedure and the risk of misplacing the injection is low. The intraperitoneal injection itself is considered a non-invasive and minimally stressful procedure for laboratory animals. Dedicated studies have proven that chronic intraperitoneal administration of saline over 30 days is well-tolerated in mice and rats [85]; however, it should be noted that injection of LPS may cause painful reactions and long-term health impairment [43]. The higher dose of LPS, namely 5 mg/kg administered once, may arise questions about animal mortality rates as this dosage is sometimes considered as “sublethal” [81]. Interestingly, a study by Lew and colleagues showed that intraperitoneal administration of high doses of LPS *E. coli* O55:B5, such as 10 and 20 mg/kg once a week, only slightly increased mortality rate of mice during first 60 days of the experiment [86]. This difference in the expected mortality rate of animals after the application of LPS at a dose of 5 mg/kg may result from the use of different types of LPS, which is discussed later in this section. Nevertheless, high doses of LPS should be considered as a potential risk factor for greater losses of tested animals. The major disadvantage and a significant limitation of LPS-induced model of neuroinflammation associated with intraperitoneal administration is its low specificity in inducing changes in the brain, which may be of importance in the case of research targeting defined anatomical structures of CNS. 

Stereotaxic LPS models such as intranigral, intrastriatal and intracerebroventricular models were intensively developed in PD studies to precisely deposit LPS within PD-associated brain regions, like SN and striatum, in order to selectively impair dopaminergic neurons and activate the microglia [9]. In a study by Lee and colleagues [78], the time needed to induce a dopaminergic denervation upon intranigral LPS administration was 4 weeks, adapted on the basis of previous experiments. After this time, the efficacy of this method was evaluated in the amphetamine-induced rotation test and immunohistological analysis of tyrosine hydroxylase expression in the SN. Both of these tests showed a significant difference between the control and LPS-injected groups. However, given the small size of the SN, LPS administered intranigrally may apply mechanical pressure to the neurons, leading to their damage. This feature can significantly interfere with the assumptions of this model by causing mechanical damage instead of changes secondary to inflammation. The striatum, as a brain structure with a larger volume, shows a lower risk of a mechanical damage in response to a direct LPS injection. Thus, the intrastriatal administration of LPS seems to be superior to the intranigral considering their application in neurodegeneration studies [9]. Deng and colleagues characterized in the detail the intrastriatal LPS model of PD [76]. They administered 10 μg of LPS unilaterally and then, after 4 and 8 weeks, behavioral tests were conducted. After 4 weeks following the surgery there was no significant difference in the rotarod test, buried food-seeking test, OFT and elevated plus maze test results between groups. However, 8 weeks after the surgery LPS-induced mice showed decreased latency time in the rotarod test, but results of other tests presented no significant difference.

The intrapallidal model is based on stereotactical administration of LPS into *globus pallidus*. However, the utilization of this model in studies is very rare, most likely due to its lower specifity in inducing SN damage comparing to intranigral and intrastriatal administration [87]. 

An intracerebroventricular administration of LPS is much more common despite of its similarly low anatomical specificity. This feature of intracerebroventricular route is actually used to induce generalized inflammation in the CNS in models of the microglia activation, cognitive impairment and AD [1]; but its deployment is less popular the research of PD [9].

In summary, stereotaxic LPS models require a specialized and expensive equipment such as stereotaxic apparatus, and the technical skill from the operating researcher [88]. It is also necessary to carefully study publications in this field in order to select specified stereotaxic coordinates. Animals exposed to this procedure must undergo general anesthesia. The surgery must be performed under strictly sterile conditions. Stereotaxic surgery causes greater traumatization in animals, which is an important aspect from the animal welfare point of view. Moreover, the anaesthesic procedures and brain surgery are associated with increased mortality rates in animals. On the other hand, intracranial LPS administration requires only one injection, compared to 5–7 shots in the intraperitoneal route. Precise methods of LPS administration such as intranigral and intrastriatal routes usually utilize rats given their larger volume of the brain and well-established coordinates for stereotaxic navigation. Nevertheless, the methods of sterotaxic LPS administration, mainly intranigral and intrapallidal, allow for the most accurate study of the LPS effects on specific parts of the brain. For example, the intrastriatal route of LPS administration is currently considered to be the most relevant LPS model to study the course of PD [88].

The intranasal LPS deposition is another example of administration route used to model PD. This method is based on PD’s underlying olfactory pathology that precedes motor dysfunction in PD patients. It has been shown that LPS administered intranasally does not cross BBB; thus, neuroinflammation is triggered by indirect pathways, similar as in other systemic models of LPS deposition [83]. The execution of this model was well-described in the latest review of Deng and colleagues [88]. In the experiment focused on PD pathology conducted by Niu and colleagues, mice were anesthetized with 2% isoflurane, and subsequently received 10 μL of LPS intranasally to each nostril once a day for 6 weeks. After the end of the LPS administration OFT, PT, the elevated plus maze test and olfactory function test were performed. Both behavioral and ex vivo tests produced results consistent with neuroinflammatory theory of PD [83]. The intranasal administration of LPS is a relatively recent method of PD modeling. It offers a new perspective on PD research, including olfactory pathology. However, this model requires administration of LPS once a day or every second day for a relatively long period of time, namely 3–20 weeks [88]. A precise intranasal deposition of a substance should be carried out under pharmacological sedation, which is also a time-consuming procedure, and may be associated with complications in anesthetized animals.

A thorough comparison of different LPS administration regimes is also offered in the review by Batista and colleagues [75]. Besides the information on LPS type, dose, route and period of administration, it also includes data on animal species used in experiments, and a summary of evaluated parameters other than behavioral tests. 

A very important aspect to consider is the bacterial origin of LPS used. Today, many LPS products are available for experimental use, including endotoxin derived from a variety of bacteria species, strains, and serotypes. The most popular bacteria species of LPS origin are *Escherichia coli, Salmonella enterica* and *Salmonella minnesota*, but there is also LPS isolated from *Porphyromonas gingivalis, Proteus mirabilis*, *Proteus vulgaris, Klebsiella pneumoniae,* and others. In addition, LPS extracted from *E. coli* is further subdivided depending on the *E. coli* strain and in the case of *Salmonella*, species division is made by serotypes. In conclusion, the variety of LPS products is large which may result in differences in their chemical structure. As mentioned in the previous section (“Lipopolysaccharide—insights in the structure”), even a small modification in the lipid A molecular composition may influence its immunogenicity and effects of its action. Unfortunately, some research papers do not provide details about utilized LPS, thus a comparison between these experiments may not be fully conclusive on the basis of solely the dose and route of administration. This issue has been recently addressed by Deng and colleagues in their latest review [88]. They reported significant differences comparing effects of systemic administration of LPS from *Salmonella abortus equi* and from *E. coli* in the same dosage.

To conclude, many of the research papers in the field of LPS-induced neuroinflammation have not been presented in this review because of insufficient information about a type of LPS used, thus making them impossible to compare. Possibly, the method of LPS purification could have impact on its immunogenic and pro-inflammatory properties as there are LPS products purified by gel-filtration chromatography, ion-exchange chromatography and phenol extracted. However, we did not find any paper describing this problem.

## 6. Therapeutic Strategies Targeting Neuroinflammation and Cognitive Dysfunction

Currently, searching for new treatment options for neurodegenerative diseases is an intensively studied topic. This focus emerges mainly from the increasing population of patients suffering from these conditions and the lack of effective therapy to inhibit or reverse progression of the disease. Numerous novel substances are being developed and tested specifically for their neuroprotective properties; however, the translation from in vitro and in vivo preclinical studies to clinical trials remains difficult. To overcome this issue, repurposing drugs already registered for other clinical purposes and testing them for their neuroprotective and memory-enhancing properties is becoming more popular. In this section, we briefly summarize reports describing the efficacy of available drugs, as a potential reference of substances influencing neuroinflammation induced by LPS. Novel potential drug candidates which are being developed for this purpose will be described in the section “Future outlook”.

### 6.1. Non-Steroidal Anti-Inflammatory Drugs and Glucocorticoids

When inflammation is involved, the choice of treatment intuitively includes anti-inflammatory drugs. Drug classes with the most pronounced anti-inflammatory effects are non-steroidal anti-inflammatory drugs (NSAIDs) and glucocorticoids (GCs). 

COX is a key enzyme involved in the inflammatory cascade of arachidonic acid. In the brain, the isozyme COX-1 is constantly present in neurons of the hippocampus and cerebral cortex, while isozyme COX-2 is expressed in microglia and neurons under pro-inflammatory stimulation [89]. NSAIDs inhibit the COX, selectively or nonselectively blocking COX-1 or COX-2. They are commonly used to relieve inflammation, pain and fever in humans and animals. The use of NSAIDs in neurodegenrative diseases is an ongoing topic. Indomethacin, a non-selective COX inhibitor, showed anti-amyloid and anti-inflammatory properties in in vitro and in vivo studies [90,91,92]. Due to such promising results, indomethacin has currently reached clinical trial phase 3 for AD treatment [89]. A COX-2 selective NSAID, namely celecoxib, has also shown good antiamnestic and anti-inflammatory results in in vivo studies utilizing the soluble Aβ-induced AD model, and has been qualified for a clinical trial for the prevention of AD [89,93]. Despite the fact that LPS also induces COX-2, there are several reports describing lack of result or even exacerbation of some LPS-induced inflammatory reactions by indomethacin, celecoxib and other NSAIDs [94,95]. Given this, it cannot be concluded with certainty that NSAIDs can be good model substances for inhibiting LPS-induced inflammation and further studies are needed to explain these controversial results being linked with LPS and NSAIDs. 

GCs, despite their broad. multi-level anti-inflammatory effect, are not proper drugs to ameliorate neuroinflammation. It is proven that chronically elevated plasma GCs levels promote AD pathomechanisms, accelerate cognitive dysfunction and are referred to as a risk factor for AD and PD [96,97]. Taking this into account, we hypothesize that in the research field CGs may be useful as agents exacerbating course of neuroinflammation and can contribute to modeling the severe course of neurodegeneration. 

### 6.2. Antidiabetic Drugs

Diabetes and insulin resistance are other known risk factors for neurodegenerative diseases, mainly because they exacerbate inflammatory processes. Therefore, insulin and antidiabetic medications have been studied, especially in AD, but their positive effects were proven only in dementia patients with concomitant diabetes [98]. However, one antidiabetic drug, namely pioglitazone, demonstrated a particularly good neuroprotective effect in animal models of dementia. According to current knowledge, this is provided by the ability of pioglitazone to bind to peroxisome proliferator-activated receptor gamma receptor (PPAR-γ) and thus inhibit proinflammatory NF-κB signaling [17,74,98]. Surprisingly, pioglitazone turned out not to be effective in the AD clinical trials [17], but its efficacy in animal models, including LPS-induced neuroinflammation, may be utilized as a reference point.

### 6.3. Dexmedetomidine

Dexmedetomidine is a sedative agent, acting as an agonist ofα_2_-adrenergic receptors. It is particularly utilized in veterinary medicine to sedate and premedicate animals. Interestingly, dexmedetomidine has been shown to have neuroprotective properties. In the animal inflammation models, including the LPS model, this drug had anti-inflammatory and antioxidant effects; moreover, it was able to reduce and even reverse neuronal apoptosis [99,100]. This led dexmedetomidine to clinical trials phase 1 for the treatment of AD [89]. These properties make dexmedetomidine a good model of a neuroprotective drug; however, when planning memory and cognition studies in animals, its pronounced sedative effect should be taken into consideration.

### 6.4. Minocycline

Another drug with prominent neuroprotective properties is minocycline. Minocycline is a second-generation semisynthetic tetracycline antibiotic with a good BBB penetration [101]. Its neuroprotective activity has been shown in various animal models of neuroinflammation including LPS model [8,102]. These protective properties on the nervous tissue are exerted by inhibiting microglial activation, reducing oxidative stress and alleviating inflammation [8,89,102]. This drug has been also qualified for clinical trials for the treatment of AD [89]. Minocycline appears to be a good candidate as a reference neuroprotective drug in the LPS-induced neuroinflammatory model, given its well-established anti-inflammatory properties, especially in the LPS model.

### 6.5. Drugs Currently Registered in AD 

Current treatment standards for AD worldwide usually include the use of cholinesterase inhibitors (namely rivastigmine, donepezil, galantamine) and memantine. These drugs are thought to provide only symptomatic treatment in AD; however, there is growing evidence that they may have additional effects on the course of the disease. Memantine is an antagonist of NMDA receptor, and it reduces the worsening of dementia symptoms by modulating glutamatergic transmission in the brain. Studies have shown that memantine could ameliorate inflammation and oxidative stress in the CNS and in the periphery [103,104], and even influence Aβ and tau pathology [89]. These effects require further studies to be fully understood; nevertheless, memantine has been already used as a comparative neuroprotective agent in the LPS-induced neuroinflammation study [8]. Cholinesterase inhibitors address the deficiencies of neurotransmitter acetylcholine observed in the AD, as cholinergic transmission is crucial for cognition, learning, and memory [105]. Similarly, memantine, rivastigmine and donepezil may also exert some neuroprotective effects, shown in a study by Kim and colleagues [106], and in other, older reports [107]. Despite an excellent paper from Kim and colleagues describing in vitro and the in vivo anti-inflammatory properties of donepezil and rivastigmine on LPS and Aβ-stimulated neuroinflammation, there is still a need to investigate other potential effects and mechanisms of action of these drugs. 

### 6.6. Monoclonal Antibodies in AD and PD 

One of the leading drug development strategies for AD is specific monoclonal antibodies against AD’s aberrant proteins, namely aducanumab and zagotenemab. Aducanumab, an anti-Aβ antibody, has been recently approved by the FDA through an accelerated approval pathway and zagotenemab, an anti-tau antibody, is being examined currently in the phase 2 clinical trials [17]. Monoclonal antibodies are also tested in the field of PD treatment research. A novel monoclonal antibody that binds aggregated α-synuclein, called prasinezumab, was assessed in the phase 2 clinical trial for its effect on PD, however its result was negative [108]. Despite this, we have not found any papers describing the use of aducanumab, zagotenemab or prasinezumab in the LPS neuroinflammatory model. However, these drugs might potentially be used in the model of LPS neuroinflammation with the aim of explaining the influence of Aβ, tau and aggregated α-synuclein pathology on its outcomes. On the other hand, the utility of these antibodies in animal experimental models might be severely limited, given they have undergone a process of humanization.

## 7. Future Outlook

### 7.1. Nature-Derived Substances

As mentioned earlier, an ideal drug for the treatment of neurodegenerative diseases is not available yet, thus big efforts are being made to develop supportive therapy based on natural substances and extracts. Many plants and animal products have been used for a very long time in the traditional folk medicine to alleviate various conditions, and this premise is often an inspiration for a closer study of their mechanism of action and pharmacological properties [41,109]. Frequently, the first step of investigating anti-inflammatory potential of these substances is the test in LPS-challenged cell lines [110]. Studies involving animal models of LPS-induced neuroinflammation are utilized to assess in vivo activity of compounds tested for this purpose for the first time. Both of these methods may be also used with the aim of explaining the exact mechanism of action and specific effects of substances with already established neuroprotective activity [41,111,112]. The most important factors limiting the therapeutic use of these substances are often their low bioavailability in humans and not sufficient level of BBB crossing. Such a limitation was shown for berberine, an alkaloid with multiple beneficial pharmacological effects including neuroprotection, which has a very limited use due to its poor pharmacokinetic properties [113].

### 7.2. Novel Compounds

Due to the continuously increasing knowledge in the fields of medicinal chemistry, pharmacology and related scientific areas, new insights into the complexity of mechanisms involved in the neuroinflammation are being gained. It enables us to develop new compounds influencing elements of that process. 

One of these elements includes spleen tyrosine-protein kinase (Syk), which modulates the neuroinflammation signaling pathway. Inhibition of Syk had positive effects in many inflammatory research models, however comprehensive influence of Syk inhibitors on neurodegenerative diseases is inconclusive yet [114]. To address this issue, Kim and colleagues [115] examined novel Syk inhibitor BAY61-3606, which stands for 2-[[7-(3,4-dimethoxyphenyl)imidazo[1,2-c]pyrimidin-5-yl]amino]pyridine-3-carboxamide, in LPS-induced in vitro and animal models. In both BV2 cell culture and in microglial cells from cortical and hippocampal areas of tested animals, BAY61-3606 attenuated expression of Syk, TNF-α, and ionized calcium-binding adaptor molecule 1 (Iba-1), which is a marker of microglia reactivation. In brains of mice receiving LPS and BAY61-3606, a reduced level of neuronal cell loss was shown in Nissl staining compared to the group treated with LPS alone. Moreover, inhibition of Syk in the brain tissue resulted in attenuation of NF-κB activity, thereby silencing a key inflammatory cascade and decreasing expression of COX-2 and iNOS, which were assessed with immunoblotting and immunofluorescence. Additionally, mRNA expression for the genes of pro-inflammatory cytokines was measured in the cell culture, and results showed that BAY61-3606 was able to prevent TNFα, Il-1β, and Il-6 genes overexpression in response to LPS. Performed behavioral tests, namely Y-maze and PAT, showed a protective effect of Syk inhibition on LPS-induced cognitive impairment [115]. This example illustrates a variety of research possibilities offered by the LPS-induced model complemented by biotechnical methods. LPS-challenged cell cultures and animal models allow for a thorough study of molecular mechanisms and pharmacological activities of drug candidates in microglia-mediated neuroinflammation. The study design described above is widely distributed in the field of novel neuroprotective compound research [116,117]. In a study by Park and colleagues, investigating properties of a selective α7 nicotinic acetylcholine receptor agonist, namely GTS-21 (3-(2,4-dimethoxybenzylidene)-anabaseine, DMXBA), cell cultures (BV2 cells and primary microglia) and mice were treated with LPS to explore GTS-21 anti-inflammatory activity [116]. Similar to the study by Kim and colleagues [115], in this experiment, levels and mRNA expression of pro-inflammatory molecules were also measured. GTS-21 influence on the of phosphorylation NF-κB, MAPK, protein kinase B (Akt) and 5'AMP-activated protein kinase (AMPK) was also examined, leading to the conclusion that GTS-21 exerts an anti-inflammatory effect by AMPK up-regulation along with Akt and NF-κB downregulation. GTS-21 also had a positive effect on anti-inflammatory transcription factors, namely cAMP-response element-binding protein (CREB) and PPAR-γ. The ability to inhibit reactivation of microglia was also assessed on the basis of a quantification of Iba-1 expression. However, a model other than LPS was used to study the neuroprotective activity of the compound in behavioral tests in mice. It demonstrates that LPS-induced models can be a part of a study combining many different research techniques, and the obtained results can complement each other. For example, screening of chemically congenial compounds in LPS-stimulated BV2 cells can be a first step to identifying candidates with the most promising anti-inflammatory activity in the microglia environment [118]. 

Based on the studies discussed above, it is noticeable that targeting the NF-κB pathway is an important determinant of anti-inflammatory effects. This is confirmed by a large amount of research focused on development of agents capable of inhibiting NF-κB, which is an early pivotal pro-inflammatory factor during the inflammatory cascade in microglia [119,120,121]. In a study by Tang and colleagues, measurement of NO production in LPS-stimulated BV2 cells was used as an aforementioned preliminary screening method for the activity of a few novel anti-inflammatory compounds [119]. Moreover, activated BV2 cells were also used in this study to examine the pivotal ability to block the TLR4/NF-κB signaling pathway. However, given the multi-level intricacy of neuroinflammation, there are many potential targets for drug discovery other than the TLR4/NF-κB pathway, for example, NLRP3 inflammasome [122,123], tyrosine phosphorylation-regulated kinase 1A (DYRK1A) [124], translocator protein (TSPO) [125], glycogen synthase kinase 3 (GSK3) [126] or protease-activated receptor 1 (PAR1) [127]. A common feature of these studies is the use of LPS in order to explore the anti-inflammatory properties of investigated compounds. It confirms that LPS-induced neuroinflammation is an important, widely distributed and ongoing research model in the field of developing and investigating new neuroprotective drugs. Nowadays computational techniques e.g., molecular docking, significantly contribute to a development of large numbers of novel compounds with promising pharmacological activity [128]. However, data obtained with in silico methods must be verified in in vitro and in vivo conditions; here, similarly to natural-based substances, LPS models can find particular application as a well-described and available research tool.

## 8. Concluding Remarks

The purified bacterial endotoxin was initially utilized in the research of endotoxic shock in order to model this directly life-threating condition in a repetitive and controlled manner. However, as our understanding of the inflammatory response and immunity broadened, it turned out that the inflammation can be a non-obvious but very important contributing factor to many other diseases, such as neurodegenerative disorders or cancer. Nowadays, LPS is used to study inflammatory response and its consequences in both in vitro and in vivo conditions, providing an insight into the pathology and creating an opportunity to develop new treatment strategies for a great variety of medical conditions, including AD and PD. Although more than half a century has passed since the first LPS research, there is still much to learn about the precise underlying molecular events contributing to LPS recognition and cellular response. TLR4 were originally thought to be the sole recognition system of LPS. TLR4 is a well-known pattern recognition receptor of the innate immunity and its role in inducing inflammatory response is very prominent. However, thanks to a lot of excellent studies, it has been established that other distinct cellular mechanisms are also involved in providing a response to endotoxin. TRP channels (TRPA1, TRPV1, TRPV4, TRPM3 and TRPM8) and caspase ‘non-canonical’ pathways (caspase 4,5 in human and caspase 11 in mice) were recently brought to light as an important LPS sensing pathways. However, their exact functions and mutual crosstalk are not yet thoroughly known, leaving some ‘white areas’ on the LPS recognition map. This field requires further detailed molecular studies; nevertheless, these pathways should be considered while obtaining and interpreting results of experiments utilizing LPS. Another important aspect that emerged during the writing of this paper was the lack of a description of exact LPS type used in some research papers. This may be a major problem for the repeatability of experiments and their utility as a base for studies of other researchers. As discussed above, even minor changes in the LPS molecule can impact its properties and immunogenicity, resulting in a significant discrepancy between induced effects and obtained results. For this reason, special attention should be paid to providing detailed information about the type of LPS used. Although LPS remains a widely distributed research model, in this paper we pointed out several areas that still remain unclear or unknown and require further studies to clarify the facts. This leads to the conclusion there is still a need to investigate and supervise even well-known research methods, such as an LPS-induced neuroinflammation model. 

## Figures and Tables

**Table 1:** A summary of different LPS administration regimens among studies utilizing LPS-induced neuroinflammation model.

Route and Period of LPS Administration	Dose	Species	Type of LPS	Performed Behavioral Tests	Reference
intraperitoneal, 7 days	500 and 750 μg/kg	mice	N/A	MWM, PAT, PT	[1]
intraperitoneal, 7 days	250, 500 and 750 μg/kg	mice	*E. coli* O111:B4	NOR, NAD, OFT	[14]
intraperitoneal, 5 days	1 mg/kg	mice	*E. coli* O26:B6 *	MWM	[70]
intraperitoneal, 7 days	250 μg/kg	mice	*E. coli* O55:B5	MWM, Y-maze test	[71]
intraperitoneal, 7 days	250 μg/kg	mice	*E. coli* O111:B4	NOR, MWM	[23]
intraperitoneal, 7 days	250 μg/kg	mice	*E. coli* O127:B8	Y-maze test, NOR, ST, OFT	[77]
intraperitoneal, single dose	5 mg/kg	mice	*E. coli* O26:B6 *	OFT, PT	[70]
intracebro- ventricular, single dose	12 μg	mice	N/A	MWM, PAT, PT	[1]
intranigral, single dose	5 μg	rats	*E. coli* O111:B4	amphetamine- induced rotation test	[78]
intrastriatal, single dose	10 µg	mice	N/A	rotarod test, buried food-seeking test, OFT	[76]
intranasal, 6 weeks	10 µg	mice	*E. coli* O55:B5	OFT, PT, olfactory function test	[79]

* In the study by Yang and colleagues, LPS from *E. coli* O26:B6 is mentioned as a material in the cell culture treatment; however, this information is not provided in the animal model-so it is hypothesized that the same LPS type has been used [70]. Abbreviations: N/A—no data available; LPS—lipopolysaccharide; MWM—Morris water maze test; PAT—passive avoidance test; PT—pole test; OFT—open field test; NOR—novel object recognition test; NAD—novel arm discrimination task; ST—splash test.

## Data Availability

Not applicable.

## Abbreviations

Aβ	amyloid-β
AD	Alzheimer’s disease
Akt	protein kinase B
AMPK	5'AMP-activated protein kinase
AP	1-activator protein-1
Ara4N	4-amino-arabinose
BAY61-3606	2-[[7-(3,4-dimethoxyphenyl)imidazo[1,2-c]pyrimidin-5-yl]amino] pyridine-3-carboxamide
BBB	blood–brain barrier
CARD	caspase recruitment domain
CD-14	cluster of differentiation-14
CD-36	cluster of differentiation-36
CGRP	calcitonin gene-related peptide
CNS	central nervous system
COX	cyclooxygenase
CR	complement receptor
CREB	cAMP-response element binding protein
C1q	complement component 1q
DAMP	damage-associated molecular pattern
DYRK1A	tyrosine phosphorylation regulated kinase 1A
FDA	Food and Drug Administration
GC	glucocorticoid
GSDMD	gasdermin-D
GSK3	glycogen synthase kinase 3
GTS-21	3-(2,4-dimethoxybenzylidene)-anabaseine, DMXBA
Iba-1	ionized calcium-binding adaptor molecule 1
IKK	Iκβ kinase complex
IKKi	IKK inducible kinase
IL	interleukin
iNOS	inducible nitric oxide synthase
IRAK	interleukin-1 receptor associated kinase
IRF	interferon regulatory factor
LBP	lipopolysaccharide-binding protein
LPS	lipopolysaccharide
LRR	leucine-rich repeat domain
MAL	MyD88- adopter-like protein
MAPK	mitogen-activated protein kinase
MIP	macrophage inflammatory protein
MWM	Morris water maze test
MyD88	myeloid differentiation primary response protein
NAD	novel arm discrimination task
NEMO	NF-κB essential modulator
NF-κB	nuclear factor-Κb
NFT	intracellular neurofibrillary tangles
NLRP3	NLR family pyrin domain containing 3
NLR	NOD-like receptor
NMDA	N-methyl-D-aspartate
NO	nitric oxide
NOR	novel object recognition task
NSAID	non-steroidal anti-inflammatory drug
OFT	open field task
PAMP	pathogen-associated molecular pattern
PAR1	protease-activated receptor 1
PAT	passive avoidance test
PD	Parkinson’s disease
PGE_2_	prostaglandin E_2_
PPAR-γ	peroxisome proliferator- activated receptor gamma
PRR	pattern recognition receptor
PT	pole test
RAGE	receptor for advanced glycation end-products
RIP1/RIPK1	receptor-interacting serine/threonine-protein kinase 1
RLR	retinoic acid inducible gene-I-like receptor
ROS	reactive oxygen species
SN	substantia nigra
SNpc	substantia nigra pars compacta
SR	scavenger receptor
SP	substance P
TAB	TAK1 binding protein
TAK1	transforming growth factor β-activated kinase 1
TBK1	TANK-binding kinase 1
TIR	Toll/IL-1 receptor domain
TIRAP	TIR domain containing adaptor protein
TNF-α	tumor necrosis factor-α
TLR	Toll-like receptor
TRAF	TNF-receptor associated factor
TRAM	TRIF-related adaptor molecule
TRIF	Toll/interleukin-1-receptor domain- containing adaptor inducing interferon β
TRP	transient receptor potential
TRPA1	transient receptor potential ankyrin 1
TRPM3	transient receptor potential melastatin 3
TRPM8	transient receptor potential melastatin 8
TRPV1	transient receptor potential cation channel subfamily V member 1
TRPV4	transient receptor potential vanilloid 4
TSPO	translocator protein
WHO	World Health Organization
